# Multi-Level Family Factors and Affective and Behavioral Symptoms of Oppositional Defiant Disorder in Chinese Children

**DOI:** 10.3389/fpsyg.2017.01123

**Published:** 2017-06-30

**Authors:** Yixin Tang, Xiuyun Lin, Peilian Chi, Qing Zhou, Xiangning Hou

**Affiliations:** ^1^Institute of Developmental Psychology, Beijing Normal UniversityBeijing, China; ^2^Department of Psychology, University of MacauMacau, China; ^3^Department of Psychology, University of California, BerkeleyBerkeley, CA, United States

**Keywords:** child with ODD, multilevel family factors, child affective ODD symptoms, child behavioral ODD symptoms

## Abstract

Given the important role of family environment in children's psychological development, the objective of this study was to examine the linkages between family factors at the whole, dyadic, and individual levels and two dimensions (affective and behavioral) of Oppositional Defiant Disorder (ODD) symptoms in Chinese children. Participants comprised of 80 father-child dyads and 169 mother-child dyads from families with ODD children. The results indicated that multilevel family factors were differently associated with children's affective and behavioral ODD symptoms. All the family factors at the dyadic and individual levels were significantly associated with child affective ODD symptoms. However, only the most proximal factors (parent-child relationship and child emotion regulation, which were directly related to child) were significantly related to child behavioral ODD symptoms. The present study extends the current knowledge regarding the relationships between family factors and two dimensions of child ODD symptoms by testing the comprehensive multilevel family factors model. This study also recommends that future interventions for ODD children should consider the multi-level family factors to enhance intervention efficacy.

## Introduction

Oppositional Defiant Disorder (ODD), one of the most commonly-occurring disorders in childhood (Egger and Angold, 2006; Lavigne et al., 2009), was defined by the DSM-5 as a recurrent pattern of angry/irritable mood, negativistic/defiant behavior, or vindictiveness toward authority figures (American Psychiatric Association, 2013). Children with ODD display persistent resistance, argumentation, and acts of aggression that disrupt interactions with peers and family members, instead of releasing occasional outbursts that result from intermittent frustration or negative events (American Psychiatric Association, 2013).

Previous studies suggested that ODD symptoms could be divided into two different dimensions, including affective and behavioral dysregulation (Burke and Loeber, 2010). Recent studies also provided empirical support for the two-dimension structure of ODD symptoms. For example, Lavigne et al. (2015) examined the metric and scalar of existing models to identify whether ODD consisted of multiple dimensions. The results suggested that the two-dimension model optimally represented the dimensionality of ODD symptoms (Lavigne et al., 2015). Symptoms of affective dimension included being touchy, short-tempered, spiteful, and resentful to others. On the other hand, symptoms of behavioral dimension included deliberately acting to annoy others, refusing to comply with majority's requests or consensus-supported rules, frequent arguing, and blaming others for their own mistakes. (American Psychiatric Association, 2013).

### Links between family factors and ODD symptoms

Previous studies have well-documented the significant linkage between family context and child psychological development (Hetherington and Martin, 1986), particularly in families with children having potential affective and behavioral problems (e.g., Cox and Paley, 2003; Smeekens et al., 2007; Lavigne et al., 2014). A wealth of literature has identified numerous family factors that placed children at increased risk of developing ODD, including poor family function, low marital quality, parental maladaptive behavior, paternal substance abuse, and low quality parent-child relationship (Burke et al., 2002; Greene et al., 2002; Marmorstein et al., 2009; Matthys and Lochman, 2010).

Both cross-sectional and longitudinal studies also proved the robust associations between family factors and child ODD symptoms. For example, Lavigne et al. (2014) found that higher scores on family risk factors (family conflict, parent hostility in parenting, child emotion temperament) were positively associated with child ODD symptoms in a cross-sectional study. Similarly, Smeekens et al. (2007) found that multiple family factors, like parent-child interaction, parent-child attachment, and various parental, child, and contextual characteristics, served as predictors of child later externalizing behavior problem in a longitudinal study.

### Three levels of family factors

Although previous studies have identified a wide range of family factors linked to child ODD symptoms, the majority of these studies focused almost exclusively on family factors at either one level or mixed levels. Different associations of multi-level family factors and child ODD symptoms remained unclear. Cox and Paley (2003) proposed that family was a dynamic and interactive system consisting of interdependent subsystems, including whole-family subsystem, parent-child subsystem, co-parenting subsystem, and marital subsystem. Factors at these subsystems served as important contexts in understanding child development (Cook and Kenny, 2006). Based on this theory, Lavigne et al. (2012) investigated the relations between family subsystems and child ODD symptoms. They proposed a multi-domain model of family factors for ODD symptoms encompassing contextual factors, parental depression, parenting, and child characteristics. Furthermore, they assessed the pathways of these multi-domain factors on child ODD symptoms. However, this model was too complex to clearly demonstrate the hierarchy of these family factors. Considering the complexity of Lavigne's model, Lin et al. (2013) proposed a concise three-level model on the basis of the Family System Theory (Cox and Paley, 2003) and the Bioecological Model (Bronfenbrenner, 2009), which delineated the mechanism of ODD development. This model divided family factors into three levels, including the whole level, the dyadic level (including couple dyadic and parent-child dyadic levels) and the individual level (including parental individual and child individual levels). In the present study, we adopted this comprehensive model to test the associations between family factors and two dimensions of ODD symptoms.

### Family factors and child's affective and behavioral problems

Previous research highlighted the crucial role of family function at the whole level in predicting children's affective and behavioral symptoms (Slee, 1996; Lucia and Breslau, 2006). Family cohesion, as one aspect of family function, was negatively correlated with child internalizing and externalizing problems (Lucia and Breslau, 2006). Children in less cohesive families were more likely to develop conduct disorder and delinquent symptoms (Leflore, 1988; Slee, 1996). In cohesive and well-adapted families, members were prone to interact with each other in a harmonious manner, which further promoted parental and children's emotion regulation abilities. Thus, children in such families were less likely to develop affective and behavioral problems.

Among the numerous dyadic level factors, marital quality, and parent-child relationship were noted as the most influential factors on children's psychological problems within the family (Lin et al., 2013). A considerable body of research has linked marital quality directly and indirectly to children's internalizing and externalizing problems (Cummings and Davies, 2002). Lower marital quality was directly related to subsequent child internalizing problems. Exposure to marital conflict was upsetting to children and appeared to elicit child maladjustment in both direct and indirect ways (Zimet and Jacob, 2001; Erath and Bierman, 2006). Additionally, poor parent-child relationship appeared to be a robust risk factor of children's behavioral adjustment (Masten and Garmezy, 1985). Negative parent–child relationships were significantly associated with child externalizing disorders (Waschbusch, 2002), namely attention-deficit/hyperactivity disorder (ADHD), conduct disorder (CD), and ODD (Burt et al., 2005). Secure parent-child relationships protected children from adverse developmental outcomes (Groh et al., 2014), whereas insecure parent-child relationship was a risk factor of child anxiety and other internalizing problems (Kerns and Brumariu, 2014).

Besides the factors at the whole and dyadic levels, specific individual characteristics of parents and children also accounted for the development of ODD symptoms (Eisenberg and Fabes, 2006; Cunningham et al., 2009). Smith (2010) suggested that parental emotion regulation played a vital role in child developmental outcomes. Parental dysregulated emotion contributed to inappropriate emotional expressions or behaviors toward children, which further predicted children's maladaptive emotional outcomes (Muhtadie et al., 2013). Moreover, parents with difficulty in emotion regulation exhibited poor emotional coaching, which would positively predict children's disruptive behavior, especially when children demonstrated high levels of emotion liability/negativity (Dunsmore et al., 2013). Previous studies also found that children's deficits in emotion regulation contributed to the manifestation of externalizing and internalizing symptoms (Southam-Gerow and Kendall, 2002; Kim and Cicchetti, 2010), whereas children with better emotion regulation demonstrated less subsequent internalizing and externalizing problems (Blandon et al., 2010; Kim and Cicchetti, 2010).

### Parenting in chinese context

Previous studies indicated that ecological context played a crucial role in shaping family functioning, parenting styles, and child development (Schleyer-Lindenmann, 2006). Currently, the majority of studies examining the associations between parenting styles and child ODD symptoms were based on Western samples. The information on how parenting in non-Western societies, such as Mainland China, affect children's psychological development remains scarce. Moreover, the implementation of the one-child policy before 2015 introduced changes into parenting styles. Some families adopted the “child-centered” approach into child-rearing, particularly among well-educated parents (Chang et al., 2003). The “child-centered” parenting style linked children and parents together closely, but it also increased the possibility that children become spoiled. Meanwhile, most parents put high expectations on their children, expecting children to be obedient, respectful, and excellent in schoolwork. Consequently, parents would be under a lot of pressure, particularly when their children misbehaved. In the present study, we examined how the specific family context and parenting styles in China contribute to children's ODD symptoms.

### The present study

We proposed a comprehensive framework concerning how multi-level family factors are differently related to children's ODD symptoms. Specifically, we included family function as the whole level family factor, marital quality, and parent-child relationship as the dyadic level family factors, and emotional regulation of both parent and child as the individual level family factors. Our study aimed to specify the different associations of the three-level family factors with child affective and behavioral oppositional defiant disorder symptoms. We hypothesized: (1) Among families with children identified with ODD, there are significant associations between three levels of family factors and children's ODD symptoms; (2) Three levels of family factors exhibit different associations with ODD symptoms. Specifically, family function, as the whole level factor and the most distal factor, is less correlated with ODD symptoms than family factors at the dyadic and individual levels. (3) Multi-level family factors exhibit different associations with two dimensions of children's ODD symptoms in that all family factors are more closely linked to affective ODD symptoms than to behavior ODD symptoms.

## Method

### Participants and sample procedure

Data in this study was derived from a large research project on family risk and protective factors of ODD children in China. Between 2013 and 2014, 14 elementary schools in northern (Beijing), eastern (Shandong province), and southwestern (Yunnan province) parts of China participated in this study. School psychologists in these 14 primary schools invited all the class master teachers who taught first grade through fifth grade to nominate the children in their classes who exhibited ODD symptoms. The nomination was based on the ODD symptoms assessment checklist, which was derived from the *Diagnostic and Statistical Manual of Mental Disorders* (DSM-IV-TR; American Psychiatric Association, 2000). In total, 187 class master teachers nominated 360 students from 7,966 children in the participating schools.

After the initial nomination, two clinical psychologists from Beijing Normal University further confirmed the assessment, using a semi-structured interview guide in interviewing class master teachers. The interview was based on the DSM-IV-TR's ODD diagnostic criteria. After the interview, two clinical psychologists discussed their assessments for each child to ensure accordance. Only children with both clinical psychologists' diagnoses of ODD were recruited into the current study. Ultimately, 305 children identified as displaying the symptoms of ODD were invited to participate in the research (3.8% of the children in the participating schools), and 282 parent-child dyads agreed to join the project. Thirty-three families were excluded from the analyses because more than 20% of the items on one questionnaire were missing.

The final ODD sample consisted of 249 parent-child dyads, including 80 father-child dyads and 169 mother-child dyads. Ages of parents ranged from 25 to 53 (paternal age *M* = 38.35, *SD* = 5.08; maternal age *M* = 36.64, *SD* = 4.28). All children (180 boys and 69 girls) aged between 6 and 13 (*M* = 9.59, *SD* = 1.59). Among these children, 80% were the only child in their families. Approximately, 141 families (56.6%) had a monthly income over 5,000 Chinese Yuan (the average monthly income for Chinese urban families is about 5,485 Chinese Yuan; National Health and Family Planning Commission of the PRC, 2015). More than half of the participants, including 151 fathers (60.6%) and 141 mothers (56.6%), reported that they completed junior college education or higher education.

### Survey procedure

After signing the informed consents, each participating child was asked to deliver a parent survey package to their father/mother. Parents were invited to fill in the parent survey and return the completed surveys to the class master teachers in 1 week.

All study procedures, including informed consent and child assent, were approved by the Institutional Review Board of Beijing Normal University. Each participant received a token of appreciation to acknowledge their participation in the study. All the 305 children who met the criteria for ODD were provided with the opportunity for treatment from psychiatrists in Beijing Anding Hospital, psychological counselors, and family therapists from the Center of Family Study and Therapy at Beijing Normal University.

### Measures

#### Two dimensions of ODD symptoms

Two dimensions of ODD Symptoms were assessed by an 8-item scale derived from the eight ODD symptoms indicated in DSM-IV-TR. This scale measures child ODD symptoms through two subscales of negative affect and oppositional behavior, each subscale includes 4 items that had the highest item total correlation coefficient (e.g., ODD affective symptoms “He/she is touchy or easily annoyed,” ODD behavioral symptoms “He argues often and blames others for their own mistakes”). Child ODD symptoms were collected in family settings. Parents evaluated children's ODD symptoms, using a dichotomous measure (0 = *no*, 1 = *yes*). A higher total score indicates more ODD symptoms. In the current study, the Cronbach's α for the negative effect and the oppositional behavior were 0.78 and 0.71.

### Whole level measures

#### Family function

Parents reported on their family function using the Family Adaptability and Cohesion Evaluation Scale (FACES-II; Olson, 2000), which has proved to be an appropriate measure for Chinese families (Chen et al., 2011). FACES-II assesses the family function in two dimensions: Adaptability (14 items; e.g., “In solving problems, the children's suggestions are followed”; α = 0.74 for this sample) and Cohesion (16 items; e.g., “Family members like to spend free time with each other”; α = 0.71 for this sample). Father/mother reported their perception of actual conditions in the family, using a 5-point scale ranging from 1 (*almost never*) to 5 (*almost always*). A higher total score on FACES-II indicated better adaptability and cohesion in the family. In the current study, the Cronbach's α for FACES-II was 0.84.

### Dyadic level measures

#### Marital quality

Parents reported on their own marital quality using the Dyadic Adjustment Scale (DAS; Spanier, 1976), which was a valid measure of marital relationship quality for Chinese couples (Shek, 1995; Gau et al., 2012). Father/mother completed four subscales of DAS: Dyadic Consensus (13 items; e.g., “Career decisions”; α was equal to 0.77), Dyadic Satisfaction (10 items; e.g., “Are you confident in your mate?”; α was equal to 0.83), Affectional Expression (4 items; e.g., “Demonstrations of affection”; α was equal to 0.60), and Dyadic Cohesion (5 items; e.g., “Work together on a project”; α was equal to 0.83). Most items of the DAS were scored using a 6-point scale, but some items were scored in 2-point and 5-point scale to better express the meaning. Higher total scores indicated higher marital quality. The Cronbach's α for the DAS total scale in the current sample was 0.90.

#### Parent-child relationship

The Parent Stress Index-Short Form (PSI-SF; Abidin, 1995) was utilized to measure the parent-child relationship. Parents were asked to rate 36 items from various aspects of their perceived interaction stress with their children, using a 5-point scale (1 = *strongly agree*, 5 = *strongly disagree*). The PSI-SF has three subscales: Parental Distress (12 items, indicating the distress resulting from personal factors such as depression or conflict with a partner and from life restrictions due to the demands of child-rearing), Parent-Child Dysfunctional Interactions (12 items, indicating parents' dissatisfaction with interactions with their children and the degree to which parents find their children unacceptable), Difficult Child Characteristics (12 items, measuring parents' perceptions of their children's self-regulatory abilities), and a Defensive Responding scale (7 items) that indicates the degree to which the parent might be attempting to deny or minimize problems. Higher scores overall indicated lower levels of parental stress and better parent-child relationship. Internal consistency was good for the PSI-SF total scale (α = 0.94).

### Individual level measures

#### Parental emotional regulation

The Difficulties in Emotion Regulation Scale (DERS; Gratz and Roemer, 2004) was used to assess parental emotion regulation ability in the present study, which has been previously used in Chinese samples (Yan et al., 2015). The DERS is a 36-item self-report questionnaire providing a comprehensive assessment of parents' overall emotion regulation difficulties as well as six specific dimensions: (a) Non-acceptance of negative emotional responses (6 items; α = 0.71), (b) Difficulties engaging in goal-directed behaviors when experiencing negative emotions (5 items; α = 0.54), (c) Difficulties controlling impulses when experiencing negative emotions (6 items; α = 0.78), (d) Limited access to emotion regulation strategies perceived as effective (8 items; α = 0.78), (e) Lack of clarity of emotional responses (α = 0.682) (5 items; α = 0.65), and (f) Lack of awareness of emotional responses (6 items; α = 0.41). The Awareness subscale was deleted from DERS in this current study because of low factor loading (β = −0.07, *p* = 0.31). Father/mother rated the items on a 5-point scale (1 = *almost never*, 5 = *almost always*). Total scores were summed for DERS, with higher scores indicating more difficulties in emotional regulation. Internal consistency was good for the DERS total scale in the current sample (α = 0.84).

#### Child emotion regulation

Children's emotion regulation ability was assessed using the Emotion Regulation Checklist (ERC; Shields and Cicchetti, 1997), which demonstrated good internal reliability in China (Chang et al., 2003). The ERC is comprised of 23 items that target processes central to children's negative emotionality and regulation. The ERC yields two subscales: Liability/Negativity subscale (15 items) and Emotion Regulation subscale (8 items). Because this study targeted the children's overall management of emotions, the Emotion Regulation subscale was used (e.g., “Can modulate excitement in emotionally arousing situations”). Items are scored on a 4-point scale ranging from 1 (*never*) to 4 (*almost always*). A higher total score was reflective of better emotion regulation of child. In the current study, the Cronbach's α of the Emotion Regulation subscale was 0.82.

### Data analysis procedures

All data in the current study was based on self-reports from parents. To reduce concerns about common method variance, we conducted Harman's one-factor test using Exploratory Factor analysis with a principal axis factoring method of extraction. The first extracted component accounted for 16.48% of the total variance, indicating that the common method variance was not of great concern (Aulakh and Gencturk, 2000).

Then, the preliminary analyses were performed to evaluate the descriptive statistics and correlations between the study variables. Preliminary data analyses were performed using SPSS 20.0. Bivariate correlations were conducted to test initial relations between variables (see Table 1).

**Table 1:** Variable descriptions and correlations among variables for children with ODD.

	***M***	***SD***	**1**	**2**	**3**	**4**	**5**	**6**	**7**	**8**	**9**	**10**	**11**	**12**	**13**	**14**	**15**	**16**
1. Family-adaptability	71.86	10.17	–															
2. Family-cohesion	48.79	7.84	0.76^**^	–														
3.PSI-parental distress	38.36	7.46	0.37^**^	0.46^**^	–													
4. PSI-dysfunction interaction	43.74	7.65	0.43^**^	0.49^**^	0.62^**^	–												
5. PSI-difficult child	37.77	8.58	0.39^**^	0.44^**^	0.57^**^	0.73^**^	–											
6. DAS-dyadic satisfaction	45.82	6.15	0.59^**^	0.44^**^	0.29^**^	0.35^**^	0.33^**^	–										
7. DAS-dyadic consensus	56.55	10.61	0.52^**^	0.45^**^	0.28^**^	0.31^**^	0.34^**^	0.64^**^	–									
8. DAS-dyadic cohesion	17.20	4.27	0.51^**^	0.43^**^	0.25^**^	0.34^**^	0.29^**^	58^**^	0.49^**^	–								
9. DAS-affective expression	11.93	2.27	0.54^**^	0.42^**^	0.31^**^	0.33^**^	0.33^**^	0.67^**^	0.69^**^	0.45^**^	–							
10. DERS-nonacceptance	11.78	4.01	−0.22^**^	−0.27^**^	−0.35^**^	−0.25^**^	−0.18^**^	−0.18^**^	−0.08	−0.13^**^	−0.15^**^	–						
11. DERS-goals	9.45	2.85	−0.32^**^	−0.33^**^	−0.32^**^	−0.33^**^	−0.16^**^	−0.22^**^	−0.17^**^	−0.22^**^	−0.23^**^	0.35^**^	–					
12. DERS-impulse	11.00	3.51	−0.35^**^	−0.36^**^	−0.41^**^	−0.37^**^	−0.28^**^	−0.20^**^	−0.12	−0.12	−0.20^**^	0.51^**^	0.57^**^	–				
13. DERS-strategies	14.81	4.50	−0.35^**^	−0.34^**^	−0.46^**^	−0.42^**^	−0.24^**^	−0.28^**^	−0.17^**^	−0.20^**^	−0.22^**^	0.58^**^	0.65^**^	0.70^**^	–			
14. DERS-clarity	9.82	2.97	−0.34^**^	−0.38^**^	−0.31^**^	−0.43^**^	−0.28^**^	−0.23^**^	−0.21^**^	−0.29^**^	−0.21^**^	0.38^**^	0.51^**^	0.50^**^	0.56^**^	–		
15. Child emotion regulation	26.34	3.50	0.33^**^	0.31^**^	0.25^**^	0.49^**^	0.40^**^	0.30^**^	0.22^**^	0.29^**^	0.26^**^	−0.28^**^	−0.25^**^	−0.35^**^	−0.40^**^	−0.44^**^	–	
16. ODD affective symptoms	1.41	1.39	−0.28^**^	−0.31^**^	−0.35^**^	−0.49^**^	−0.62^**^	−0.20^**^	−0.16^**^	−0.14^*^	−0.15^*^	0.19^**^	0.11	0.22^**^	0.21^**^	0.17^**^	−0.43^**^	–
17. ODD behavioral symptoms	1.23	1.33	−0.31^**^	−0.35^**^	−0.43^**^	−0.54^**^	−0.64^**^	−0.23^**^	−0.23^**^	−0.22^**^	−0.20^**^	0.21^**^	0.16^**^	0.28^**^	0.31^**^	0.24^**^	−0.44^**^	0.71^**^

* p < 0.05;

** *p < 0.01*.

**Figure 1:**
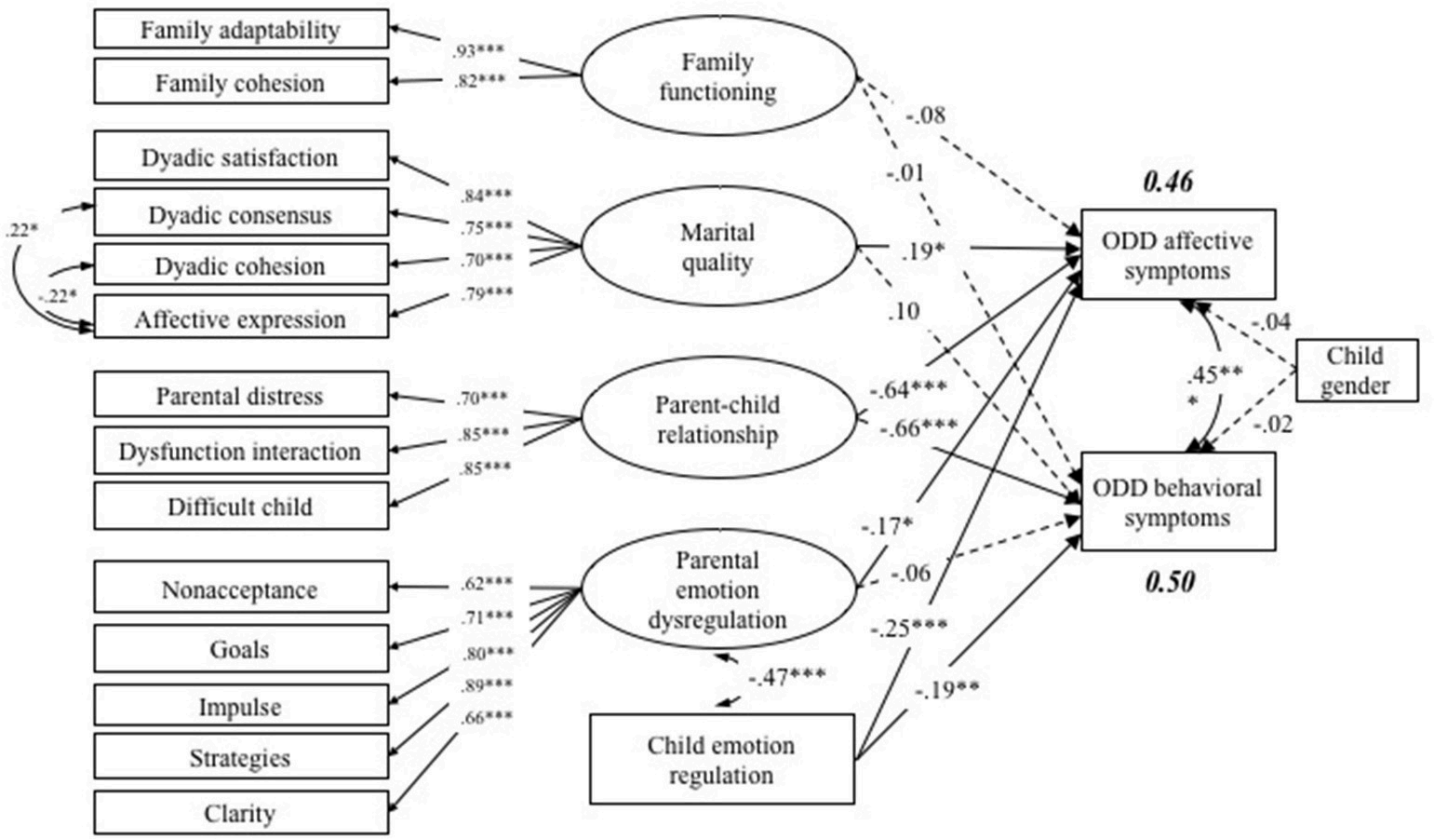
Model of multilevel family factors associated with ODD affective and behavioral symptoms. This is a standardized model, ^*^p < 0.05, ^**^p < 0.01, ^***^p < 0.001. The interrupted line means the path is not statistically significant.

Finally, the structural equation model (SEM, see Figure 1) was tested using Mplus Version 7.0 (Muthé and Muthén, 2010). Model fit criteria used in this study were chi-square statistic (χ^2^), the goodness-of-fit index (CFI), the Tucker-Lewis index (TLI), the root mean square error of approximation (RMSEA), and the standardized root mean residual (SRMR). A model is typically considered as acceptable fit the data when the CFI and TLI values are larger than 0.90, the RMSEA value < 0.08, and the SRMR value is no > 0.08 (Hu and Bentler, 1999).

## Results

### Preliminary analysis

Descriptive statistics and bivariate correlational analyses were presented in Table 1. As shown in Table 1, basically each dimension of family function, marital quality, parent-child relationship, parent emotion regulation, child emotion regulation, and ODD symptoms was significantly correlated with each other (*ps* < 0.01).

### SEM analysis

Before testing the hypothesized structural model, the preliminary analyses examined the gender, primary school grade, number of siblings, as well as household income differences in two dimensions of child oppositional defiant disorder symptoms (see Table 2). Among all these demographic variables, only the children's gender was significantly associated with observed variables. Hence, children's gender was placed into the subsequent path model test as covariates, yet it did not show any significant effect in the models presented below.

**Table 2:** The characteristics of children with ODD.

	**ODD affective symptoms *M* (*SD*)**	**ODD behavioral symptoms *M* (*SD*)**
**CHILD GENDER**		
Boy (*n* = 180)	1.53(1.37)	1.35(1.34)
Girl (*n* = 69)	1.09(1.40)	0.91(1.28)
*F*	5.24^*^	5.50^*^
η^2^	0.03	0.02
**SIBLINGS**		
Only child (*n* = 199)	1.46(1.38)	1.24(1.31)
One or more sibling (*n* = 50)	1.22(1.40)	1.19(1.42)
*F*	1.17	0.06
η^2^	0.00	0.00
**FAMILY INCOME**		
<5,000 RMB per month (*n* = 112)	1.26(1.39)	1.17(1.33)
Over 5,000 RMB per month (*n* = 137)	1.53(1.38)	1.28(1.34)
*F*	2.38	0.36
η^2^	0.01	0.00
**CHILD PRIMARY SCHOOL GRADE**		
Junior grade (*n* = 70)	1.64(1.39)	1.36(1.36)
Middle grade (*n* = 117)	1.41(1.33)	1.22(1.30)
Upper grade (*n* = 62)	1.15(1.46)	1.11(1.37)
*F*	2.08	0.59
η^2^	0.02	0.00

* *p < 0.05*.

Junior grade, 1st and 2nd grade in primary school;

Middle grade, 3rd and 4th grade in primary school;

*Upper grade, 5th and 6th grade in primary school*.

Table 2 presented the means and standard deviations for ODD affective and behavioral symptoms across basic demographic variables. Further, ANOVA was conducted to compare two dimensions of child ODD symptoms in various demographic groups. There was no significant difference of child affective ODD symptoms [*F*_(1, 247)_ = 1.17, *p* = 0.28] and child behavioral ODD symptoms [*F*_(1, 247)_ = 0.06, *p* = 0.81] between families with only one child and families with siblings. Families with different monthly incomes did not show significant difference in reporting child affective ODD symptoms [*F*_(1, 247)_ = 2.38, *p* = 0.12] and child behavioral ODD symptoms [*F*_(1, 247)_ = 0.36, *p* = 0.55]. Children from different primary grades did not score differently on child affective ODD symptoms [*F*_(2, 246)_ = 2.08, *p* = 0.13] and child behavioral symptoms [*F*_(2, 246)_ = 0.59, *p* = 0.56]. However, it is noteworthy that there were significant differences in boy's and girl's affective and behavioral ODD symptoms. Hence, children's gender was placed into the subsequent model test as covariates, yet it did not show significant effect on either dimension of children's ODD symptoms in the model presented below. A model of proposed relationships among the study variables, controlling for child gender, was presented in Figure 1.

Model results indicated that the proposed model fit the data very well (χ^2^ = 236.23, *df* = 114, CFI = 0.95, TLI = 0.93, RMSEA = 0.06). Results of the model indicated that on the whole level, family function was not significantly associated with either dimensions of child ODD symptoms. When testing the association of dyadic level factors on child ODD symptoms, we found that both marital quality (β = 0.19, *p* < 0.05) and parent-child relationship (β = −0.64, *p* < 0.001) were significantly linked to the ODD affective symptoms. However, only the parent-child relationship was significantly related to the ODD behavioral symptoms (β = −0.66, *p* < 0.001). At the individual level, both parental emotion regulation (β = −0.17, *p* < 0.05) and child emotion regulation (β = −0.25, *p* < 0.001) were significantly correlated with child ODD affective symptoms, but only child emotion regulation was negatively linked to ODD behavioral symptoms (β = −0.19, *p* < 0.01).

## Discussion

The current study tested the multilevel family factors model proposed by Lin et al. (2013), trying to provide a comprehensive understanding of the associations between multilevel family factors and two dimensions of child ODD symptoms. Specifically, the current study examined family factors at the whole, dyadic, and individual levels in explaining child ODD behavioral and affective symptoms. Consistent with our hypotheses, three levels of family factors showed different associations with affective and behavioral ODD symptoms in Chinese Mainland families. Family factors at the whole level were less correlated with ODD symptoms than factors at the dyadic and individual levels. Also, multi-level family factors exhibited different associations with two dimensions of child ODD symptoms in that all family factors were more closely linked to affective ODD symptoms. The present study highlighted the value of studying child affective and behavioral ODD symptoms in the family context.

Model results indicated that multilevel family factors were differently associated with child ODD symptoms. The findings in the current study attracted our attention to the distal and proximal ends of the socio-biological environment in child ODD development (Lin et al., 2013). As the most distal factor, family function was not significantly associated with either dimension of child ODD symptoms. This finding was consistent with our hypothesis, as well as findings of previous studies (e.g., Grant et al., 2006). Representing the wholeness and higher order of family environment, family function was not directly related to child psychopathological outcomes (Grant et al., 2006). In the proximal end of family environment, dyadic and individual levels family factors exhibited stronger correlation with child ODD symptoms than the distal factors. All the factors in the dyadic and individual levels were significantly linked to child affective ODD symptoms, but only the most proximal factors (the factors directly related to children—parent-child relationship and child emotion regulation) showed significant linkages with child behavioral symptoms. The findings indicated that the family factors in the proximal end of family environment exhibited robust association with child ODD symptoms. The family factors in the distal end of family environment might exert its effect indirectly, through its effect on the proximal factors, on child psychological development (Grant et al., 2006).

The findings in the current study also indicated that family factors were more significantly related to children's affective ODD symptoms than to children's behavioral ODD symptoms. All the family factors at the dyadic and individual levels exhibited significant associations with child affective ODD symptoms, but only the most proximal factors (the factors directly related to children—parent-child relationship and child emotion regulation) showed significant association with child behavioral ODD symptoms. The result indicated that child affective ODD symptoms were more susceptible to family factors than child behavioral ODD symptoms. It was explainable that the behavioral problems served as the outer form of child affective problems, which were usually motivated by affective problems (Carver and Scheier, 2004; Aldao and Christensen, 2015). Deficits in emotion regulatory abilities were known to contribute to the manifestation of externalizing symptoms (Southam-Gerow and Kendall, 2002; Yap et al., 2007) Additionally, a network analysis of ODD symptoms also suggested that affective symptoms appeared to be relatively central to the homogenous ODD symptoms network while behavioral symptoms fell along the periphery (Smith et al., 2016). For children with ODD, affective ODD symptoms placed them at a greater risk of developing into ODD behavioral symptoms. Family risk factors exerted effects on child affective ODD symptoms, and went further on child behavioral ODD symptoms. Aggravation of affective symptoms might lead to further deterioration of the overall ODD symptoms.

The findings of the current study demonstrated the different associations between family factors and two dimensions of child ODD symptoms. Making sense of these findings, future intervention should focus on family factors at the dyadic and individual levels, which might firstly decrease child affective ODD symptoms and further decrease child behavioral ODD symptoms. The different associations of family factors with child affective and behavioral ODD symptoms in the current study were generally consistent with previous literature in that ODD symptoms consisted of two separate dimensions. This finding further lend credit to the two-dimension structure of ODD symptoms (Lavigne et al., 2012). ODD symptoms should be divided into two separate dimensions that differentially depict affective and behavioral ODD symptoms.

Several limitations of the current study should be addressed when interpreting the data. First, all data in the current study was based on self-reports from the child's father/mother. Although the Harman's one-factor test indicated that the common method variance was not of great concern, further study should try different measures. Second, this study adopted a cross-sectional study method; therefore, causal relationships could not be established from this study. The associations between family factors and child ODD symptoms were also very likely to be bi-directional and transactional, the multi-level family factors might not be paratactic as well. Distal family factors (e.g., family function) might predict dyadic level factors (e.g., marital relationship and parent-child relationship) and further influence parental and children's individual characteristics (e.g., parental and child emotion regulation), which would finally result in ODD. Further research with longitudinal design is encouraged to examine the direction of the associations between multilevel family factors and child ODD symptoms and the potential mediating effect. Some paths (marital quality and parental emotion regulation to child affective and behavioral ODD symptoms) in the SEM model also exhibited opposite patterns with the correlation results. Currently the underlying mechanism remains unclear, further studies are needed to explore the underlying mechanism.

Despite these limitations, the current study provided a comprehensive understanding of the associations between multilevel family factors and two dimensions of child ODD symptoms. It also provided useful applications for intervention in decreasing children's ODD symptoms from the perspective of multilevel family environment. Instead of focusing solely on parent-child interaction, which was widely adopted in most interventions for ODD (Lavigne et al., 2012), understanding child affective and behavioral ODD symptoms in the broader family context is critical to develop the best possible interventions for child ODD. Additionally, professionals should consider the type of child ODD symptoms and the different associations between multilevel family factors and affective and behavioral oppositional defiant disorder symptoms when designing interventions. If a child exhibits affective oppositional defiant disorder symptoms, interventions and counseling strategies should focus more on strengthening parent-child relationships, marital relationship, and improving parent and child emotion regulation. On the other hand, if a child exhibits behavioral oppositional defiant disorder symptoms, interventions and counseling strategies should focus more on strengthening parent-child relationships and improving child emotion regulation.

## Author contributions

Each of the five authors contributed a lot to the current manuscript. YT: Substantial contributions to the conception or design of the work; the acquisition, analysis, and interpretation of data for the work; drafting the work and revising it critically for important intellectual content; XL: Substantial contributions to the conception or design of the work; revising the draft critically for important intellectual content; PC, QZ, and XH: editing the whole paper and revising the language; ensuring the work are appropriately investigated and resolved. Each author read the final version of the current manuscript and support its submission.

### Conflict of interest statement

The authors declare that the research was conducted in the absence of any commercial or financial relationships that could be construed as a potential conflict of interest.
